# The Curious Case of Elevated Tryptase: Workup and Differential in Family of Four

**DOI:** 10.7759/cureus.38065

**Published:** 2023-04-24

**Authors:** Audra L Cochran, Christopher Coop, Brittanie I Neaves, Stuart T Wood

**Affiliations:** 1 Internal Medicine, Keesler Medical Center, Biloxi, USA; 2 Allergy and Immunology, Keesler Medical Center, Biloxi, USA; 3 Infectious Disease, Keesler Medical Center, Biloxi, USA

**Keywords:** systemic mastocytosis, hereditary alpha tryptasemia, elevated tryptase, familial, tryptase, mast cell disorder, mast cell activation syndrome, mcas

## Abstract

Elevated basal serum tryptase (BST) levels are markers of both mast cell activation and overall mast cell burden. We present a family of four individuals with elevated tryptase levels greater than or equal to 20 mcg/L, all of whom exhibited signs and symptoms suggestive of mast cell activation. Differential diagnoses included hereditary alpha tryptasemia (HaT), systemic mastocytosis (SM), and mast cell activation syndrome (MCAS). In three individuals, SM was ruled out with normal morphology on bone marrow biopsy combined with negative genetic markers. Further workup would be required for the diagnosis of MCAS since serum tryptase levels were not obtained in our emergency department during acute episodes. Although genetic testing for HaT was not available upon initial workup, HaT remains the most likely explanation for this family’s elevated BST.

## Introduction

Elevated basal serum tryptase (BST) levels can be found in up to 6% of the general population, although the clinical significance of this finding is currently unclear. Tryptase is an enzyme found both on the surface and within mast cells and basophils. When mast cell degranulation occurs, mature tryptases stored in mast cell secretory granules are released, constituting a type I hypersensitivity reaction. The most common cause of elevated BST is hereditary alpha tryptasemia (HaT), whereas systemic mastocytosis (SM) and mast cell activation syndrome (MCAS) are much rarer [1]. Here we present a familial cohort of four individuals with elevated tryptase levels and corresponding symptoms typically associated with mast cell activation.

## Case presentation

**Figure 1:**
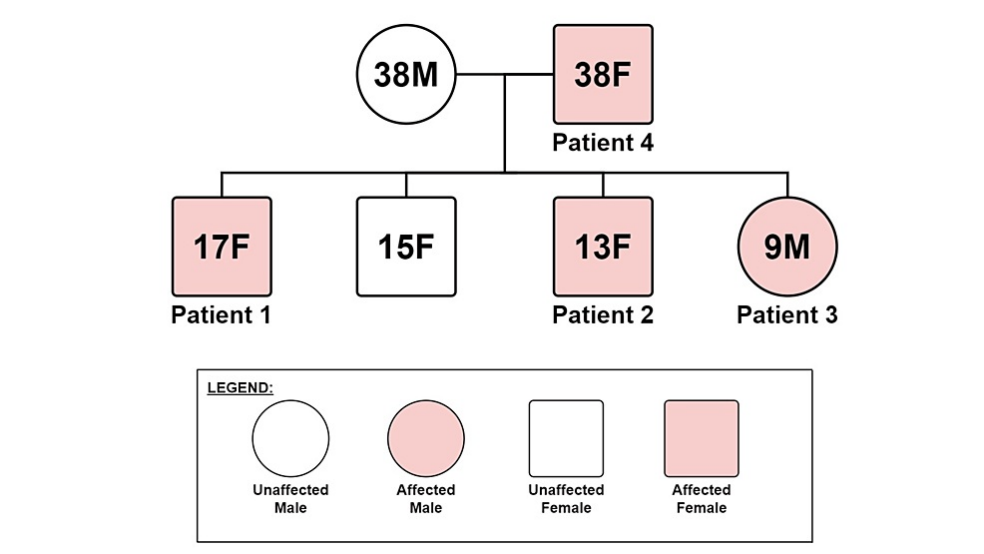
Family Tree

Our family comprised four affected individuals, a mother and three of her four children (Figure 1). The father and one of the children were unaffected. Informed oral consent was given for all four patients by patient four, the mother of patients one, two, and three.

**Figure 2:**
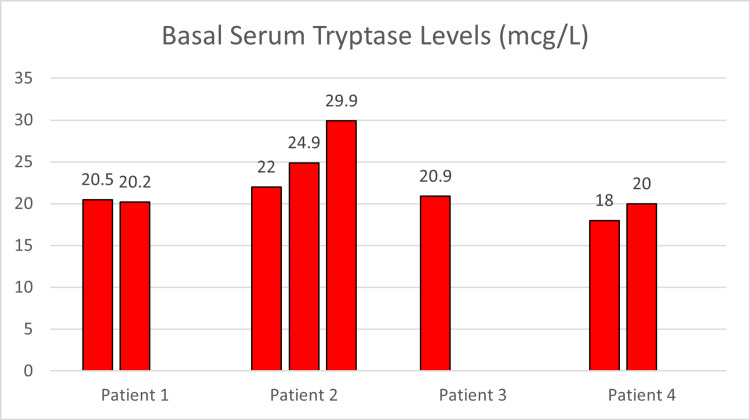
Basal Serum Tryptase Levels Y-axis represents basal serum tryptase levels in mcg/L. X-axis corresponds to patient identification. mcg/L: micrograms per liter

Patient one was a 17-year-old female with a medical history notable for asthma, seasonal allergic rhinitis, postural orthostatic tachycardia syndrome (POTS), migraine headaches, dyspepsia, obstructive sleep apnea, chronic urticaria, and episodic facial angioedema. She had elevated BST levels of 20.5 mcg/L and 20.2 mcg/L (Figure 2). Genetic testing for KIT D816V and other KIT variants (whole gene sequencing for deletions and duplicates) was negative in patient one.

Chronic urticaria and episodic facial angioedema began at five years of age in patient one. She had a resolution of these symptoms after being placed on diphenhydramine. At 10 years of age, she began experiencing episodes of severe fatigue, migraine headaches, and difficulty with concentration. At 14 years of age, she had an acute episode of slurred speech, confusion, and difficulty concentrating. Magnetic resonance imaging (MRI) of the brain was unremarkable at the time. The tilt table test was positive and she was diagnosed with POTS, with subsequent control of her symptoms after initiating fludrocortisone and metoprolol. Upper endoscopy was performed with a diagnosis of eosinophilic esophagitis, gastritis, and duodenitis. The patient was started on esomeprazole and a budesonide oral suspension with a resolution of her dyspepsia. Allergy skin testing was positive for sensitivity to pollens, cat dander, and dog dander. Her rhinitis, chronic urticaria, and episodic facial angioedema were controlled after the initiation of cetirizine. Asthma, diagnosed by spirometry, was controlled with the administration of fluticasone/salmeterol in combination with the use of an albuterol rescue inhaler.

A bone marrow biopsy in patient one demonstrated normal marrow cellularity without an increase in the number of mast cells in addition to negative mast cell CD2 and CD25 expression and negative tryptase staining (Table 1). A complete blood count, thyroid function tests, and a metabolic panel were all within normal limits.

**Table 1:** Bone Marrow Biopsy Results TNP: test not performed

Patient ID	Bone Marrow Cellularity	Tryptase Staining
Patient 1	Normal	Negative
Patient 2	Normal	Negative
Patient 3	TNP	TNP
Patient 4	Normal	Negative

Patient two was a 13-year-old female with anaphylactic episodes concerning MCAS and the sister of patient one. She had a history of chronic urticaria and episodic facial angioedema (controlled on cetirizine), non-allergic rhinitis, gastroesophageal reflux (controlled on pantoprazole), and idiopathic anaphylaxis (IA) (given on-hand epinephrine autoinjector). Her anaphylaxis episodes involved dyspnea, flushing, abdominal cramping, and urticaria.

Her tryptase levels were elevated at 22 mcg/L, 24.9 mcg/L, and 29.9 mcg/L (Figure 2). Bone marrow biopsy showed normal marrow cellularity without an increase in the number of mast cells in addition to negative mast cell CD2 and CD25 expression and negative tryptase staining (Table 1). Specific IgE testing on trees, grasses, weeds, molds, cats, dogs, and dust mites was all negative. Spirometry, a complete blood count, a thyroid function test, and a metabolic panel were within normal limits.

Patient three was a nine-year-old male, the brother of patients one and two, and the son of patient four. He had a history of episodic flushing and urticaria, both controlled with cetirizine. Tryptase level was measured as 20.9 mcg/L (Figure 1) and a complete blood count was unremarkable. No bone marrow biopsy was performed and thus the diagnosis of SM couldn’t be conclusively ruled out. Genetic testing for KIT D816V and other KIT variants (whole gene sequencing for deletions and duplicates) was negative in patient three.

Patient four was a 38-year-old female also with MCAS. She was the mother of the other three patients. She had a history of chronic urticaria, intermittent episodes of flushing, migraine headaches, gastroesophageal reflux, non-allergic rhinitis, IA, and acute episodes of confusion. These conditions had been well controlled with cetirizine, famotidine, sumatriptan, and an on-hand epinephrine autoinjector. Her laboratory evaluation was remarkable for tryptase levels of 18 and 20 mcg/L (Figure 1). Specific IgE testing for multiple aeroallergens was negative. A complete blood count, a metabolic panel, and a thyroid function test were all normal. A bone marrow biopsy demonstrated normal marrow cellularity, negative mast cell CD2 and CD25 expression, and negative tryptase staining (Table 1).

## Discussion

The differential for elevated BST is broad and includes disorders such as SM, MCAS, and HaT [2].

SM is hematopoietic cancer caused by the uncontrolled proliferation of mutated mast cells, resulting in an increased total quantity of these mutated mast cells. The World Health Organization describes major and minor criteria required to establish the diagnosis of SM. For instance, the presence of mast cell clusters in the bone marrow represents a major criterion. Minor diagnostic criteria include the presence of the KIT D816V mutation, elevated serum tryptase, and CD25 expression on mast cells [3]. SM was definitively ruled out in patients one, two, and four based on bone marrow biopsy with no mast cell clusters and no aberrant mast cells (Figure 2).

MCAS is a condition with a normal quantity of mast cells that are overactive from a functional standpoint. MCAS was first described as a distinct entity in 2010 and has remained relatively rare. To make the diagnosis of MCAS as per the updated 2021 Vienna consensus criteria, three conditions must be met: (1) acute multisystemic symptoms consistent with mast cell activation; (2) an event-related increase in serum tryptase above the serum baseline tryptase (SBT), according to the tryptase formula: 20% of SBT plus 2 ng/mL (nanograms per milliliter, which is equivalent to micrograms per liter); and (3) an appropriate response to medications targeting mast cell activation with mediator release. The symptoms of MCAS are due to excessive mast cell mediator release and involve at least two of the following four organ systems: cutaneous (e.g. flushing, pruritis, acute urticaria, angioedema), gastrointestinal (e.g., abdominal cramping, nausea, vomiting, diarrhea), respiratory (e.g., shortness of breath, nasal congestion, rhinorrhea, wheezing), and cardiovascular (e.g., hypotension and syncope) [4].

Since our family’s tryptase levels were all obtained at baseline, it is unknown as to whether or not their serum tryptase levels would have been increased during acute episodes, so the diagnosis of MCAS could not be definitively made nor excluded without further workup. Patient two’s serum tryptase levels met the tryptase formula threshold (e.g., from 22 mcg/L to an increase of at least 20% of 22 plus 2, or 6.4 mcg/L, since 29.9 is >28.4; Figure 2), however, this could not be attributed to mast cell activation since she was not acutely symptomatic when either of the tryptases was drawn.

HaT is an autosomal dominant trait present in an estimated 4-6% of the general population. HaT is a genetic condition caused by extra copies of the TPSAB1 gene, which codes for alpha tryptase. Interestingly, the increase in SBT levels in HaT is greater than would be expected based on gene replication alone. This increase in serum basal tryptase with HaT is thought to occur due to increased synthesis of pro-alpha-tryptase rather than as a consequence of mast cell activation. Not only is there a higher incidence of HaT in patients with SM and IA, but the presence of HaT portends a more severe clinical phenotype in both conditions. Curiously, only about one-third of HaT patients are symptomatic [5].

Given the high prevalence of HaT in the general population, it seems most likely that our family would be affected by HaT rather than other differentials. Unfortunately, we were unable to obtain genetic testing for TPSABS1 since this testing was not available at our facility even as a send-out, per our geneticist.

The TPSAB1 gene encoding α-tryptase has been associated with higher BST levels as well as a higher incidence of MCAS [6]. Sabato et al. reported a monogenic form of hypertryptasemia with autosomal dominant inheritance in seven relatives of three consecutive generations. These individuals had MCAS with basal tryptase levels greater than 20 ng/mL [7]. Finally, variable genetic alterations in KIT were detected in two pedigree families at either position 816 of the amino acid sequence or at other sites of KIT in systemic MCAS [8].

Our case series features a family with multiple symptoms of mast cell activation and highlights multiple practical barriers to workup, such as genetic testing for TPSABS1 not being routinely available and serum tryptase not being ordered in the emergency department when patient two sustained an episode of anaphylaxis. Four members of this family have symptoms associated with mast cell activation along with elevated BST levels.

SM was ruled out in patients one, two, and four via bone marrow biopsy. We were unable to evaluate for MCAS since serum tryptase levels were not obtained in our emergency department during acute episodes. Our top differential for this family is HaT, but we were unable to obtain genetic testing for HaT at our facility. The clinical utility of genetic testing for HaT is relatively low since the results would not alter management, and larger studies are needed to establish the role of genetic testing for HaT in the context of elevated BST [9]. Nevertheless, HaT testing would be interesting to explore from an academic standpoint and would likely be pursued should it become available at our facility in the future. The logistical obstacles in the workup of this family are likely commonly encountered issues in clinical practice. Fortunately, the limitations in these patients’ workups have no bearing on their treatment, and all four of these individuals have responded well to medical therapies.

## Conclusions

In conclusion, we report a familial cohort of four individuals with elevated BST and suspicion of HaT. This case series is unique in that all members of the family with elevated BST were symptomatic, which would be a relatively unusual presentation of HaT. The limitations in diagnostic workups are likely commonly encountered given that availability of genetic testing for HaT is relatively limited and rarely performed. Fortunately, all four of these individuals have responded well to medical treatment. It is important for clinicians to be aware of the differential for elevated serum tryptase as more primary care providers are ordering tryptase levels.
